# Research hotspots of irisin in the nervous system: a bibliometric study and visualization analysis via CiteSpace

**DOI:** 10.3389/fnagi.2025.1488559

**Published:** 2025-05-19

**Authors:** Jiacheng Wang, Juan Liu, Wei Pang, Xin Li, Xinping Huang, Shan Wang, Chunxiao Nan

**Affiliations:** ^1^College of Rehabilitation Medicine, Jiamusi University, Jiamusi, Heilongjiang, China; ^2^Children Rehabilitation Nerve Laboratory, Jiamusi University, Jiamusi, Heilongjiang, China; ^3^The Third Affiliated Hospital of Jiamusi University, Jiamusi, Heilongjiang, China; ^4^Ningbo Rehabilitation Hospital, Ningbo, Zhejiang, China

**Keywords:** irisin, nervous system, neuroprotection, CiteSpace, visualization analysis, bibliometric study

## Abstract

**Objective:**

To visually analyze the current status, hotspots, and frontiers of irisin in the nervous system and to provide a reference for future clinical research.

**Methods:**

Web of Science Core Collection (WoSCC), PubMed, Embase, and Cochrane Library databases were searched for studies on the mechanism of irisin on the nervous system published from January 1st, 2012 to April 15th, 2025. CiteSpace 6.4.R1 and Excel 2021 software were used to visualize the knowledge map of the annual number of publications, research institutions, countries, co-cited literature, journal dual map overlays, and keywords of the sample literature.

**Results:**

Four hundred and ninety-eight were included, and the annual number of articles showed an overall upward trend. The institution with the most significant number of articles was the Universidade Federal do Rio de Janeiro (UFRJ), and the country with the most significant number of articles was China. The prevalent keywords were “Exercise,” “Skeletal muscle,” “Neurotrophic factor,” “Expression,” “Alzheimer’s disease,” indicating that the production of irisin and its protective mechanisms for the nervous system are research hotspots in this field. Cluster analysis showed that the research frontier hotspots in this field could focus on three main topics: the functional role and mechanism of irisin, pathological experimental research, and clinical disease research.

**Conclusion:**

In recent years, research on irisin in the nervous system has primarily centered on elucidating its neuronal regulatory mechanisms and neuroprotective effects, along with their potential applications in neurological disorders. Future investigations should aim to delineate novel signaling pathways and action mechanisms and systematically identify therapeutic targets for refractory neurological conditions through comprehensive translational studies.

## 1 Introduction

Irisin is a new muscle cytokine discovered by Professor Pontus Boström’s team in 2012 and first reported in “Nature” (Boström et al., 2012). Irisin is a splicing and secretory fragment of fibronectin type III domain-containing 5 (FNDC5) protein, which is regulated by peroxisome proliferators-activated receptorγcoactivator-1α (PGC-1α) and secreted from muscle to blood. It promotes the transformation of white fat into brown fat with the ability to decompose metabolic fat, thereby regulating energy metabolism and reducing insulin resistance. In recent years, studies have shown that irisin is not only strongly expressed in skeletal muscle (Boström et al., 2012), but also strongly expressed in various regions of the brain tissue (Dun et al., 2013). Irisin may act as a neurotrophic factor to promote the survival, maintenance and function of neuronal cells (Zhang and Zhang, 2016), and has been confirmed in brain injury caused by cerebral ischemia (Li et al., 2017), stroke (Liu et al., 2020) and anxiety (Uysal et al., 2018). Irisin has become a new therapeutic factor that can improve cognitive, learning and memory functions. It not only regulates critical physiological processes including neural differentiation and neuroendocrine homeostasis but also mediates pathological progression in neurological disorders, demonstrating promising translational potential for clinical interventions (Waseem et al., 2022).

CiteSpace software is an information visualization software and an interactive analysis tool. Visualization in scientific maps can be achieved through the combination of bibliometrics, visual analysis methods and data mining algorithms. CiteSpace software tools can help users to quickly grasp frontier directions and hot issues in a scientific field, to discover underlying knowledge bases, and to critically examine research literature, ultimately to identify leading researchers and key institutions (Chen and Song, 2019).

In this study, CiteSpace software was used to visualize the research on the mechanism of irisin on the nervous system in the Web of Science core collection database.

## 2 Data and methods

### 2.1 Data source and retrieval strategy

To ensure a comprehensive and targeted literature search, we meticulously adopted a retrieval strategy that combines subject terms and free terms. This approach enabled us to capture both the core concepts and related peripheral topics within the research domain. Subsequently, we conducted a systematic search in the Web of Science Core Collection (WoSCC), PubMed, Embase, and Cochrane Library databases. Taking the search formula applied to the Web of Science Core Collection as an example: TS = (Irisin OR FNDC5) AND TS = (nervous system OR brain function OR neurobehavioral effects OR neuroprotection OR nervous system diseases OR neurological diseases OR Alzheimer’s disease OR Parkinson’s disease OR cerebral ischemia). No disciplinary filtering was applied to ensure the coverage of interdisciplinary literature. The researchers involved in this study verified the effectiveness of the strategy through reverse retrieval of known highly relevant literature, ensuring that the coverage rate of core literature is greater than 90%. The time range for this retrieval was precisely set from January 1st, 2012 to April 15th, 2025, in order to cover the most relevant and up-to-date academic publications for our study.

### 2.2 Inclusion and exclusion criteria

#### 2.2.1 Inclusion criteria

1. Literature that meets the application of irisin in nervous system research; and 2. Literature types were research papers, reviews, systematic reviews, and Meta-analysis.

#### 2.2.2 Exclusion criteria

1. Literature types: book chapters, editorial materials, conference abstracts, letters, news, briefs, etc.; 2. Study on the mechanism of irisin on other systems, such as musculoskeletal system diseases and endocrine system diseases; and 3. Study the mechanism of action of similar therapeutic factors on the nervous system, such as the role of new candidate brown adipokine (Batokine) in central nervous system diseases.

### 2.3 Literature screening and data extraction

**Figure 1:**
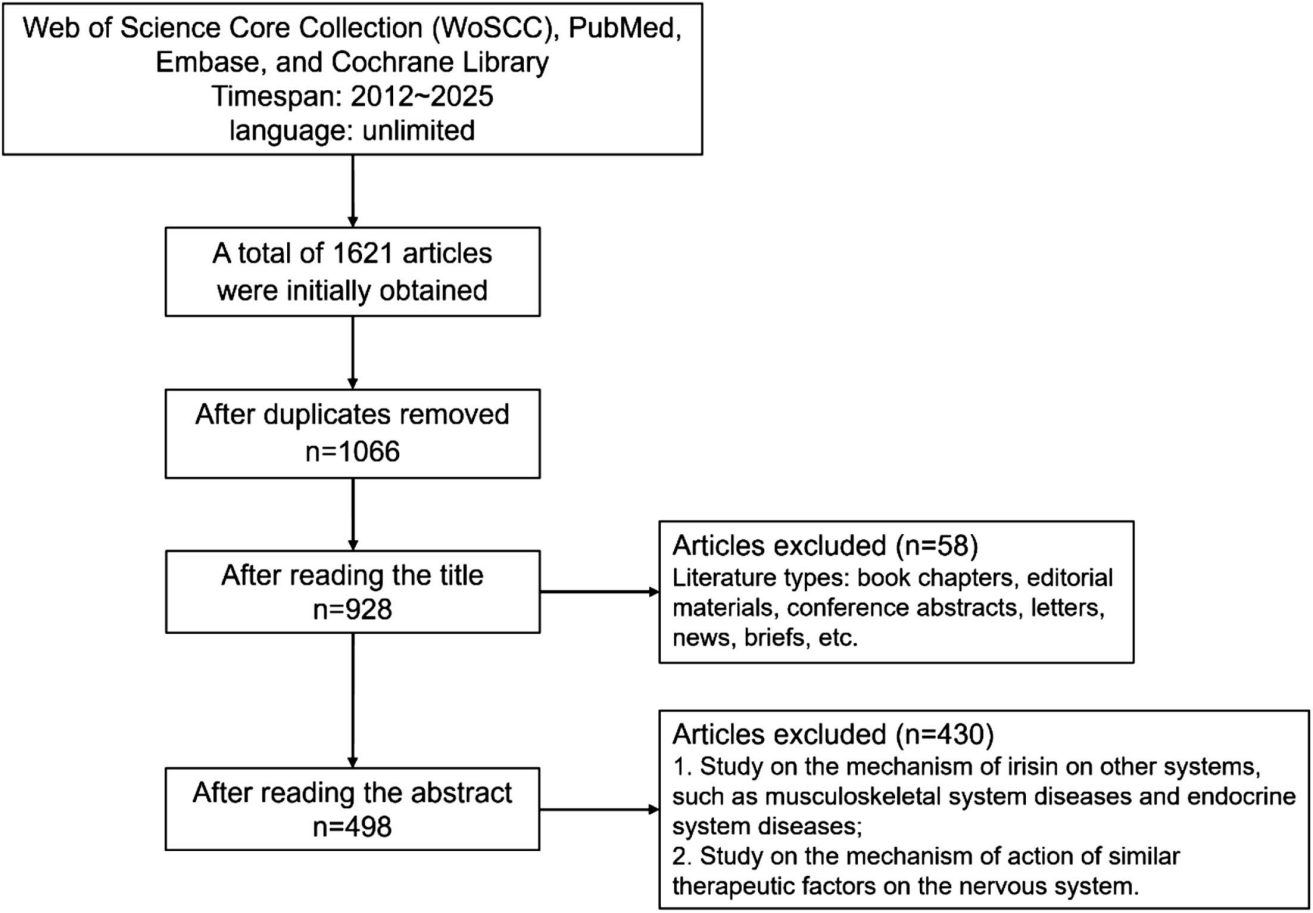
Flowchart of literature collection. The literature screening process was rigorously conducted in strict adherence to predefined inclusion and exclusion criteria.

Combining Web of Science Core Collection (WoSCC) with PubMed, Embase, and Cochrane Library enhances systematic review comprehensiveness. While WoSCC emphasizes high-impact multidisciplinary journals, it lacks depth in biomedical and pharmacological research. PubMed supplements MEDLINE-indexed biomedical literature, Embase adds drug studies, conference abstracts, and EMTREE-indexed terminology, and Cochrane integrates rigorous evidence from trials and systematic reviews. This multi-database approach minimizes selection bias and improves reliability of evidence synthesis. At the inception of the research, an exhaustive search was conducted in the above-mentioned databases, and a total of 1,621 articles were ultimately retrieved. In the ensuing screening echelon, in strict compliance with the pre-defined inclusion and exclusion benchmarks, articles that were unambiguously extraneous to the research theme were methodically expunged. This entailed a painstaking review modus operandi, wherein the titles, abstracts, and keywords of each literary piece were scrupulously perused and dissected for the screening imperative. Subsequent to a succession of stringent screening protocols, a total of 498 high-caliber research articles were ultimately incorporated into the final analysis, thereby guaranteeing the reliability and pertinence of the research sample (Figure 1).

The titles included in the literature were exported and saved in plain text file format. The recorded content was “full record and cited references”, named “download_1-498.txt” format, and the visual analysis map was drawn using CiteSpace software.

#### 2.3.1 Parameter setting

Time Slicing was set to 2012–2025; the Years Per Slice was set to “1” year; title, Abstract, Author Keyword (DE), and Keyword Plus (ID) in Term Source are all checked. Select the node type (Node Types): Institution, Country, Reference, Keyword: Association strength, other thresholds to keep the system default; the node selection methods g-index, Top *N*, and Top *N* % were set on demand according to the node type selected above. At the same time, Excel 2021 software was used to make the trend chart of annual publication volume.

## 3 Results

### 3.1 Temporal analysis of annual publication volume

**Figure 2:**
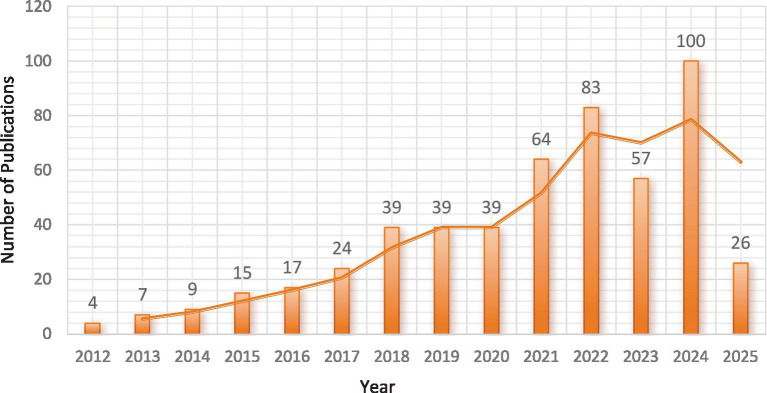
Analysis of annual publication trends on irisin in the nervous system.

As shown in Figure 2, Excel 2021 software was used to draw the trend chart of annual publication volume. The annual publication volume of irisin research in the field of the nervous system showed a spiral upward trend. The first research literature appeared in 2012, and the research field was in its infancy in 2018 and before. The annual publication volume was no more than 39, increasing rapidly from 2019. Although it decreased slightly in 2023, it showed an upward trend. The highest number of publications was 100 in 2024. It shows that researchers worldwide are making more and more explorations and attempts in nervous system research. At the same time, the rapid growth in recent years shows that there are still many unsolved problems in this research field to be studied, and the research prospects are broad.

### 3.2 Bibliometric analysis of publishing institutions

**Figure 3:**
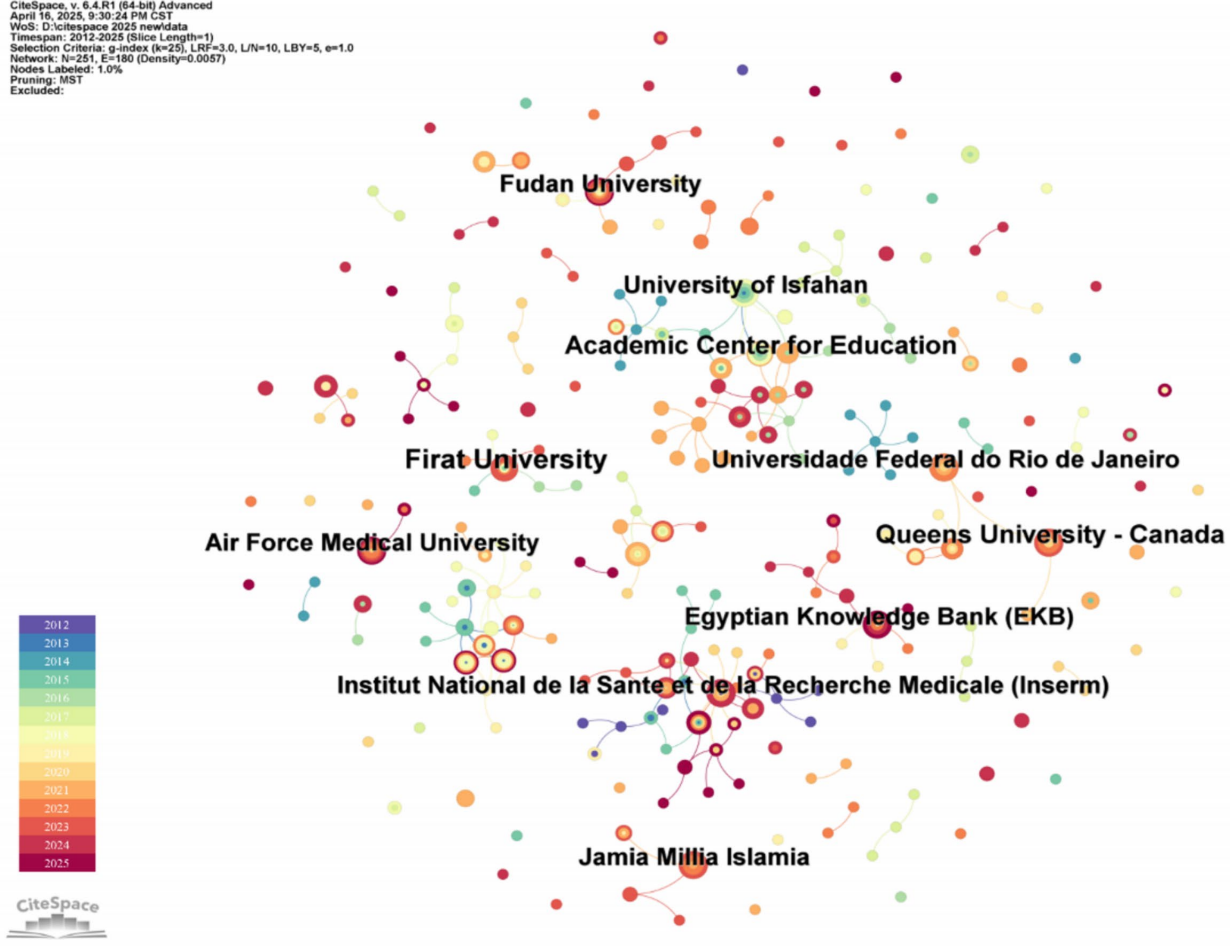
Institutional collaboration network map of irisin in the nervous system. The size of the nodes represents the number of publications, and the number of connecting lines indicates the cooperation frequency.

As shown in Figure 3, “Institution” is selected for analysis on CiteSpace software, and a map with node *N* = 251 and a number of connections E = 180 was obtained. Table 1 lists the top eight institutions with the most significant number of publications, of which the largest is Universidade Federal do Rio de Janeiro (UFRJ) in Brazil. The inconsistent standardization of institutional affiliations in the Web of Science Core Collection database leads to insufficient statistical recognition of institutional contributions by CiteSpace algorithms. To address this limitation, our study implements a comprehensive analysis of high-impact publications and co-author institutional networks, thereby enhancing bibliometric evaluations of publishing institutions. However, the bibliometric analysis in the remaining sections of this paper does not address this issue. Influenced by geographical factors such as countries and regions, collaborating institutions predominantly comprise universities, affiliated hospitals, and research organizations within the same region. For instance, among the top eight institutions with the highest publication output are the Iranian Education and Academic Center and the University of Isfahan. Additionally, a team of researchers from Brazil’s UFRJ (Prof. M.V. Lourenço, S.T. Ferreira, and F.G. De Felice) have made substantial contributions to this research field.

**Table 1:** Top eight institutions in the number of publications.

Rank	Institution	Country	Publication number	Main year
1	Universidade Federal do Rio de Janeiro (UFRJ)	Brazil	12	2021
2	Firat University	Turkey	10	2014
3	Academic Center for Education	Iran	8	2013
4	Jamia Millia Islamia	India	8	2021
5	Air Force Military Medical University	China	7	2020
6	Gdansk University of Physical Education & Sport	Poland	7	2017
7	University of Isfahan	Iran	7	2013
8	Institut National de la Sante et de la Recherche Medicale (Inserm)	France	7	2012

### 3.3 Bibliometric analysis of country/regional contributions

**Figure 4:**
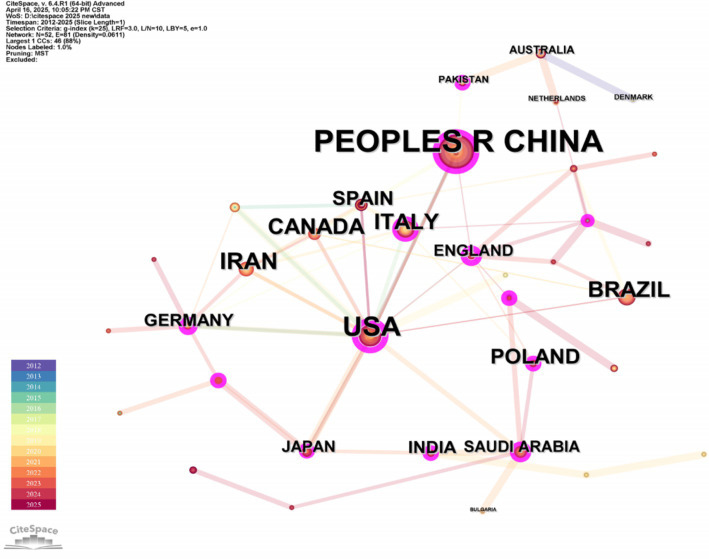
Country/region co-occurrence network map of irisin in the nervous system. It presents the modified network visualization, with the top five centrality-ranked countries/regions highlighted for improved visual clarity.

As shown in Figure 4, “Country” was selected for analysis on the CiteSpace software, and a graph with the number of nodes *N* = 52 and the number of connections E = 81 was obtained. Table 2 shows the top 10 countries in terms of the number of published papers. The top five countries in terms of the number of published papers are China (102), USA (60), Iran (28), Italy (25), and Canada (25). The top five countries in the centrality ranking are the USA (0.59), China (0.22), Italy (0.16), Poland (0.14), and Republic of Korea (0.09). The USA and China are at the forefront of the number of publications and centrality rankings and have more connections with other countries/regions, indicating that they play an important role in the research field of irisin in the nervous system.

**Table 2:** Number of publications by country/region.

Rank	Country/region	Number of publications	Centrality ranking	Year
1	China	102	0.22	2015
2	USA	60	0.59	2012
3	Iran	28	0.01	2013
4	Italy	25	0.16	2016
5	Canada	25	0.04	2014
6	Brazil	23	0.03	2019
7	Poland	20	0.14	2015
8	Turkey	19	0	2014
9	Republic of Korea	17	0.09	2017
10	Spain	15	0.04	2013

### 3.4 Bibliometric analysis of co-citation reference

**Figure 5:**
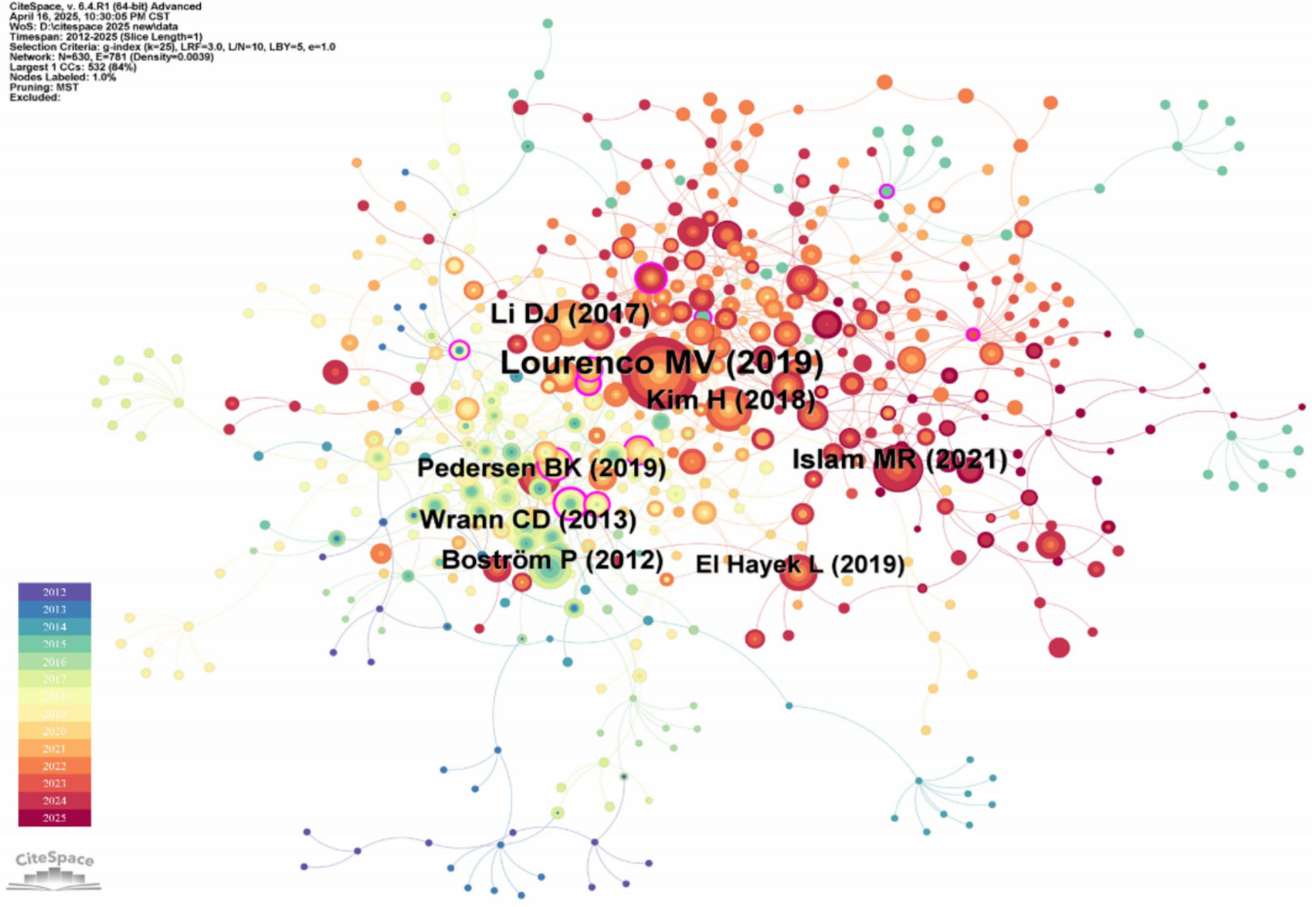
Document co-citation network map of irisin in the nervous system. This network topology maps intellectual structures within a research domain by analyzing pairwise citation relationships among scholarly documents. Nodes represent cited references, scaled by their citation frequency, while edges denote co-citation strength, with thickness or chromatic gradients proportional to the frequency of documents being cited together.

The co-citation relationship of the literature reflects the close correlation between the research directions or research topics. The more frequently the two articles are co-cited, the more robust the correlation between their academic research directions. High-frequency cited and high-center literature can reflect the research hotspots and frontier issues in this field. As shown in Figure 5, the “Reference” is selected for analysis on the CiteSpace software, and the number of nodes *N* = 630 and the number of connections E = 781 are obtained.

The top five literature of co-cited literature frequency and co-cited literature centrality are shown in Tables 3, 4. The research article of Lourenco et al. (2019) has the highest citation frequency. By demonstrating that FNDC5/irisin is an essential medium for the beneficial role of exercise in Alzheimer’s disease models, Lourenco MV et al.’s research uses FNDC5/irisin as a new drug that can fight synaptic failure and memory disorders in Alzheimer’s disease. In recent years, the therapeutic mechanism of irisin for Alzheimer’s disease has been repeatedly cited and mentioned, which can be considered a research hotspot of irisin in the nervous system. In the highly central research article of the cited literature, Professor Peng J revealed for the first time that irisin reduced oxygen–glucose deprivation (OGD) -induced neuronal damage by inhibiting the ROS-NLRP3 inflammatory signaling pathway (Peng et al., 2017). He found that irisin-induced treatment may have a regulatory mechanism in ischemic stroke and strongly influence this research field.

**Table 3:** Top five co-cited articles in frequency ranking.

Co-cited literature	Type	Number of citations
Lourenco et al. (2019)	Research article	126
Li et al. (2017)	Research article	55
Islam et al. (2017)	Research article	54
Kim et al. (2018)	Research article	49
Boström et al. (2012)	Research article	45

**Table 4:** Top five co-cited articles ranked by centrality.

Co-cited literature	Year	Centrality
Lee et al. (2014)	2014	0.35
Chen et al. (2017)	2017	0.22
Moon et al. (2016)	2016	0.19
Jedrychowski et al. (2015)	2015	0.17
Petrovic et al. (2010)	2010	0.16

### 3.5 Journal dual-map overlay analysis

The Dual Map Overlay Theory, by integrating the dual perspectives of citation and being cited, offers a unique interdisciplinary dynamic visualization tool for the analysis of scientific literature. It is particularly suitable for gaining a global understanding of complex research ecosystems and formulating strategic plans (Chen and Leydesdorff, 2014). Its core value lies in transforming the abstract citation relationships into intuitive spatial trajectories, enabling researchers, policymakers, and institutional managers to more efficiently comprehend the mechanisms of scientific knowledge production and dissemination.

**Figure 6:**
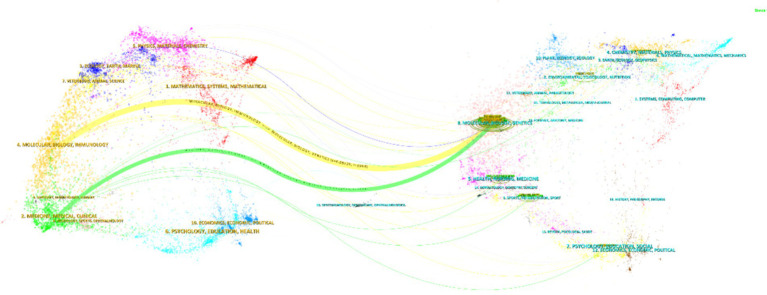
Journal dual map overlays of irisin in the nervous system. This visualization employs a dual-map framework to simultaneously analyze the disciplinary distribution of scholarly journals (left map) and their citation relationships (right map). Each node represents a journal cluster, positioned based on topic similarity derived from bibliographic coupling or co-citation networks.

The set of cited journals depicted on the left side of the figure represents the forefront of irisin research in the field of neuroscience. In contrast, on the right side, based on the disciplines of the cited literature, the basic research landscape of irisin in the field of neuroscience is established. This analysis identified two primary citation trajectories. The yellow trajectory indicates that papers published in *Molecular/Biology/Genetics* (z = 6.88235, *f* = 8,998) have exerted a significant impact on journals in the field of *Molecular/Biology/Immunology*. Likewise, the green trajectory highlights the extent to which publications in the *Medicine/Medical Care/Clinical* field have been influenced by the insights from the *Molecular/Biology/Genetics* discipline (z = 2.1654525, *f* = 3,088). The prediction based on the journal dual map overlay suggests that the research hotspots and trends of irisin in the nervous system will diverge from *Molecular/Biology/Genetics* to the two fields mentioned above (Figure 6).

### 3.6 Bibliometric analysis of keyword co-occurrence networks

#### 3.6.1 Keyword co-occurrence network analysis

**Figure 7:**
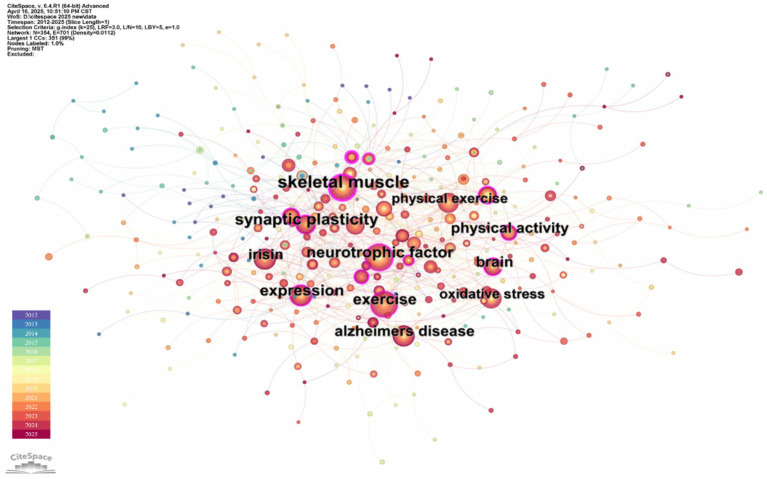
Keyword co-occurrence network map of irisin in the nervous system. The visualization illustrates the top nine high-frequency keywords, where nodes represent keywords with sizes directly proportional to their occurrence frequency (i.e., larger nodes denote more central terms in the field). Edges connect co-occurring keywords, with thickness or color saturation corresponding to co-occurrence strength (higher frequency pairs exhibit stronger visual emphasis).

Keywords are a highly condensed version of the characteristics of scientific research, reflecting the hot spots in this field and the relationship between them. The co-occurrence of keywords can inform the latest research trends and possible future directions (Sabé et al., 2023; Liu et al., 2023). As shown in Figure 7, “Keyword” is selected as the node for co-occurrence analysis on CiteSpace software, and the map with the number of nodes *N* = 354 and the number of connections E = 701 is obtained.

**Figure 8:**
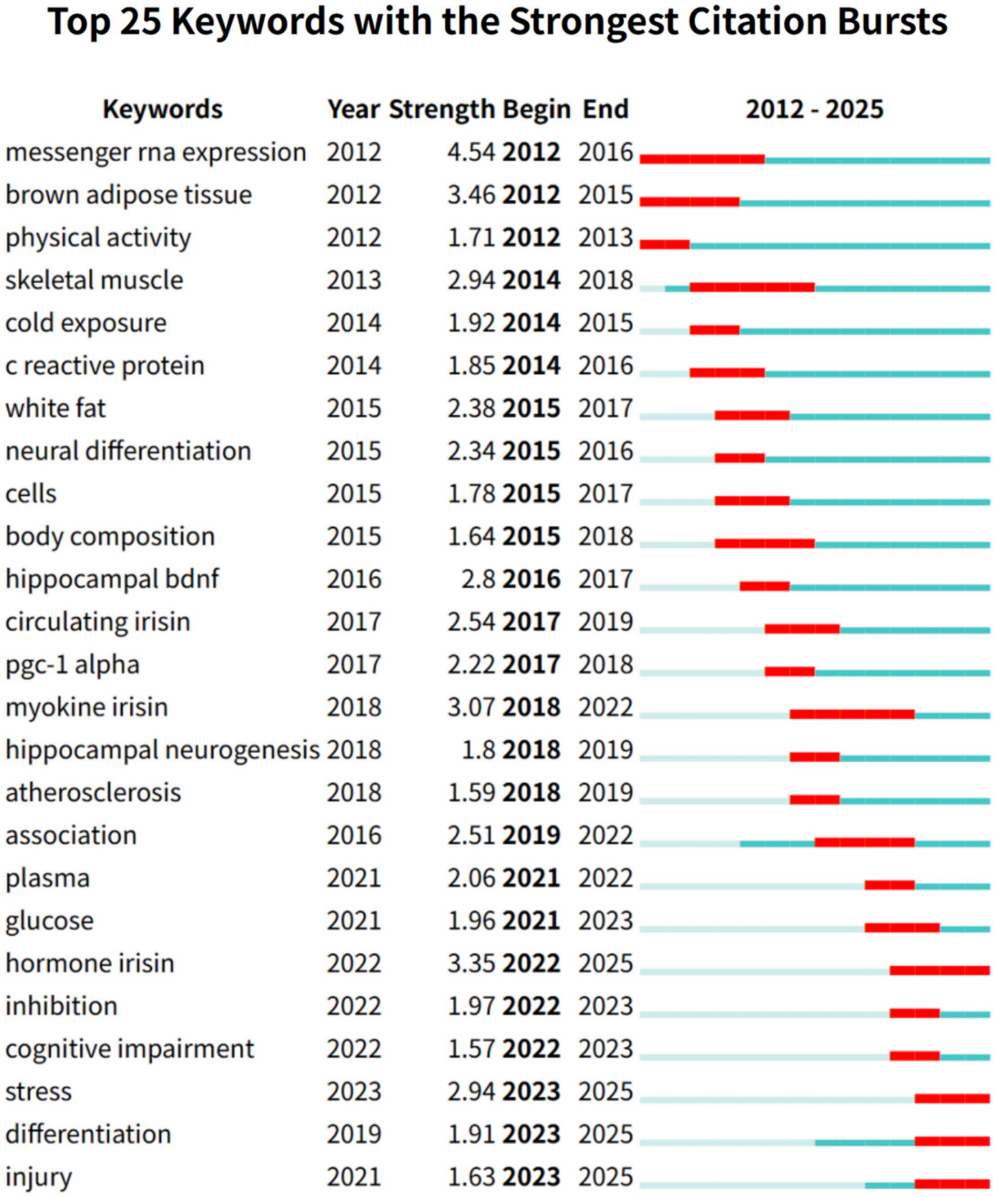
Keyword burst map of irisin in the nervous system. This visualization illustrates the temporal dynamics and frequency of keywords associated with irisin within the context of the nervous system. The blue line demarcates the overarching time interval under analysis, framing the study period. The red line highlights periodic intervals when the keywords exhibit heightened activity or prominence, reflecting bursts of research focus or discovery.

There were 14 keywords with a frequency of ≥30 times, of which the top five keywords were “Exercise” (88 times), “Skeletal muscle” (73 times), “Neurotrophic factor” (68 times), “Expression” (60 times), “Alzheimer’s disease” (55 times). The top five centrality rankings were “Skeletal muscle” (0.28), “Gene expression” (0.21), “Adipose tissue” (0.17), “Expression” (0.15), and “Synaptic plasticity” (0.15). “Exercise” and “Skeletal muscle” are the keywords with the highest co-occurrence frequency and centrality, respectively, indicating that the production mechanism of irisin has always been a research hotspot and direction of experts and scholars worldwide. The current study suggests that irisin, a new type of muscle cytokine, is produced by skeletal muscle during exercise, especially aerobic exercise. The muscle cytokine irisin released by motor muscles affects the expression of brain-derived neurotrophic factor synthesis in the hippocampal dentate gyrus (Phillips et al., 2014). It is worth noting that, as shown in the keyword burst analysis in Figure 8, the blue line indicates the time interval and the red line indicates the periodic time when the keywords are found. The keywords “Messenger RNA expression,” “Brown adipose tissue” appeared in 2012 with the discovery of irisin, and “Skeletal muscle” (2014–2018) also appeared earlier and lasted for a long time, indicating that it has shown a more lasting and stable interest in the early stage. In contrast, the keywords “Hormone irisin,” “Inhibition,” and “Differentiation” have appeared more frequently in the past 3 years, indicating that the exploration of irisin in the nervous system is more and more focused on the molecular level and signaling pathways.

#### 3.6.2 Keyword cluster analysis

Keyword clustering is an interconnected network cluster formed by keywords with themes similar to those in the research field. The connotation of each cluster is identified by the title words used frequently in the article (Sabe et al., 2022).

**Figure 9:**
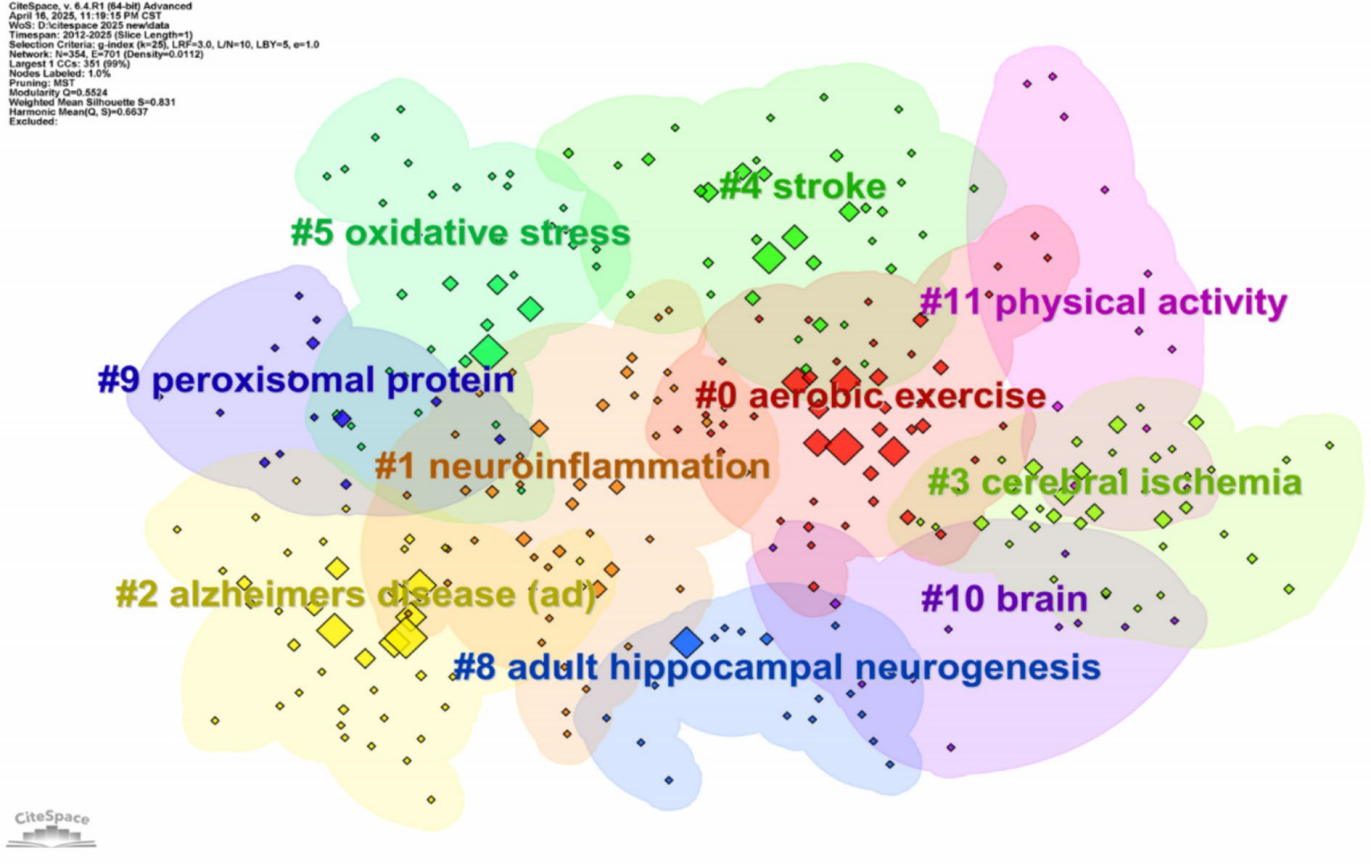
Keyword cluster network map of irisin in the nervous system. This visualization maps scholarly literature through co-citation relationships. Node size reflects citation frequency, edge thickness indicates co-citation strength, and spatial layout groups conceptually linked works. It identifies core foundational papers and emerging interdisciplinary connections within a research domain.

According to the network structure and the clarity of clustering, CiteSpace provides two indicators: module value (Q value) and average contour value (S value). Generally speaking, the Q value is typically in the interval of (0, 1), and Q > 0.3 means that the divided community structure is significant. When the S value is 0.7, clustering is highly efficient and convincing. If it is above 0.5, clustering is generally considered reasonable (Wang et al., 2023). Based on the keyword co-occurrence network, the LLR algorithm is used to cluster the keywords in the literature, and the clustering labels with clustering IDs of # 0 to # 10 are displayed. The visualization results are shown in Figure 9, and the module value Q = 0.5524 > 0.3, the average contour value S = 0.831 > 0.7, and the clustering results are significantly credible.

The identified 11 clusters (Table 5) were categorized into three major themes: the functional mechanisms of irisin, pathological experimental approaches, and clinical neurological diseases. However, a more in-depth exploration of these classifications is necessary to assess their alignment with established research paradigms and their potential to uncover new insights.

**Table 5:** Clustering table of keywords.

Cluster ID	Size	Silhouette	Mean (year)	Label
0	46	0.813	2018	Aerobic exercise
1	45	0.797	2019	Neuroinflammation
2	42	0.762	2018	Alzheimer’s disease (AD)
3	38	0.734	2018	Cerebral ischemia
4	36	0.799	2021	Stroke
5	36	0.916	2015	Oxidative stress
6	24	0.879	2017	Whole-body plethysmography
7	21	0.908	2019	Neuronal plasticity
8	18	0.861	2018	Adult hippocampal neurogenesis
9	14	0.803	2015	Peroxisomal protein
10	12	0.936	2019	Distress disorder

Cluster #0 Aerobic exercise, #5 Oxidative stress, #7 Neuronal plasticity, and #8 Adult hippocampal neurogenesis highlight the role of irisin in exercise-induced neuroprotection and synaptic regulation. While these themes align with existing literature, the prominence of #5 Oxidative stress and #7 Neuronal plasticity in recent years suggests a shift in research focus toward molecular and signaling pathway exploration. Notably, the co-occurrence of “aerobic exercise” and “adult hippocampal neurogenesis” underscores the well-documented mechanistic link between physical activity and brain health.

Clusters #6 Whole-body plethysmography and #9 Peroxisomal protein reflect methodological and signaling pathway foci. The inclusion of “whole-body plethysmography” as a high-impact cluster may indicate a growing emphasis on translational animal models for studying irisin’s systemic effects. However, this cluster’s low centrality and sparse connections to clinical keywords (e.g., “biomarkers” or “therapeutic efficacy”) suggest limited integration between preclinical and clinical research, a critical gap requiring future attention.

Clusters #2 Alzheimer’s disease, #3 Cerebral ischemia, and #4 Stroke dominate clinical research, consistent with irisin’s neuroprotective potential in neurodegeneration and cerebrovascular disorders. Meantime shows that Alzheimer’s disease, cerebral ischemia, and so on are the research hotspots of irisin in nervous system diseases.

## 4 Discussion

As a novel myokine, irisin has remained a focal point of scientific inquiry since its discovery by Boström et al. (2012), with research intensity demonstrating a consistent upward trajectory over the subsequent 13-year period. Pre-2017 investigations established its involvement in diverse physiological and pathological processes, particularly its regulatory functions in metabolic disorders, including obesity and diabetes mellitus. Subsequently, emerging evidence demonstrated the widespread presence of FNDC5 mRNA and FNDC5/irisin immunoreactivity across multiple cerebral regions (Zhang and Zhang, 2016). This study represents the inaugural bibliometric investigation and visual analysis examining irisin’s neurological implications, systematically evaluating its dual roles in neural system homeostasis and pathogenesis while proposing strategic directions for future research endeavors.

### 4.1 Current research landscape and frontier hotspots of irisin in neural system physiology

#### 4.1.1 Neuronal development

**Figure 10:**
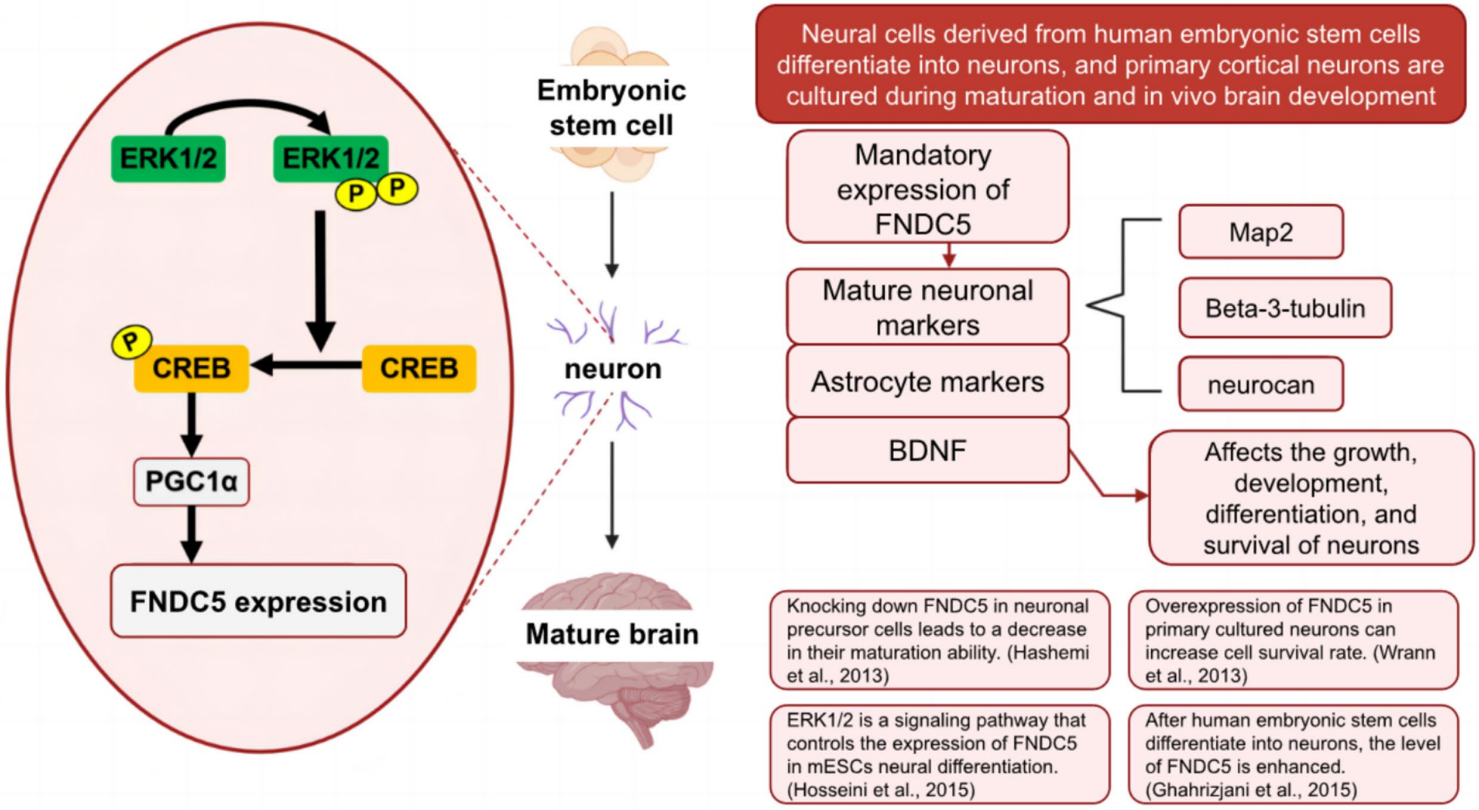
Mechanistic diagram of irisin/FNDC5 in neuronal development.

Irisin/FNDC5 can promote the expression of neurotrophic factors such as brain-derived neurotrophic factor (BDNF) and play an essential role in promoting neurodevelopment, a hotspot in this research field. As shown in Figure 10, the level of FNDC5 was enhanced during the differentiation of human embryonic stem cell-derived nerve cells into neurons (Ghahrizjani et al., 2015) and during the maturation of cultured primary cortical neurons and *in vivo* brain development. Forced expression of FNDC5 during the formation of neural precursor cells in mouse embryonic stem cells can promote the synthesis of mature neuronal markers (Map2, b-tubulin III, and neuroproteoglycan), astrocyte markers (GFAP), and BDNF (Islam et al., 2017). At the same time, Hashemi et al. found that knockout of FNDC5 in neuronal precursors impaired its development in mature neurons (and astrocytes), indicating the developmental role of FNDC5 in neurons (Hashemi et al., 2013).

However, clinical correlation remains elusive, as current human data are limited to observational studies. Firstly, analysis of RNA sequencing data from 2,114 samples of 1,234 human subjects showed that the expression of FNDC5 in the parahippocampal gyrus of AD patients was significantly decreased compared with the control group, but this difference was not observed in other brain regions (Islam et al., 2021). Secondly, intervention scalability is constrained by the short plasma half-life and limited blood–brain barrier permeability of recombinant irisin in primate trials. Addressing these bottlenecks requires standardized irisin bioactivity assays and phase II trials evaluating combinatorial delivery strategies like nanoparticle encapsulation.

#### 4.1.2 Signaling pathway

In the keyword clustering timeline analysis, the keywords in the clustering # 12 Signaling pathway appeared more in the past 3 years, indicating that the signal pathway research of irisin on the nervous system mechanism has been the research hotspot of scholars in the past 3 years. The research field of signaling pathways can be divided into the neuroprotective effect of irisin and the promoting effect of neural differentiation.

Firstly, the related signaling pathways revealed irisin’s role in promoting neurons’ growth and development. Hosseini Farahabadi et al. (2015) found that FNDC5, PGC1-*α*, and BDNF are the downstream targets of the ERK1/2 signaling pathway. They preliminarily described the signaling pathway that controls FNDC5 expression during neural differentiation of mouse embryonic stem cells (mESCs). Secondly, irisin can reduce neuronal damage caused by cerebral ischemia by activating Akt and ERK1/2 signaling pathways and help promote the neuroprotective effects of physical exercise (Li et al., 2017; Gmiąt et al., 2018). The neuroprotective effect of irisin may also be attributed to anti-inflammatory effects. Irisin inhibits the expression of pro-inflammatory cytokines (IL-6, IL-1*β*, and TNF-α) by targeting the MAPK signaling pathway and enhancing the anti-inflammatory cytokine IL-10 (Yu et al., 2022). In addition, irisin can prevent oxidative stress-induced neuronal damage. Exogenous irisin significantly increased the expression of BDNF and UCP2 and reduced the level of reactive oxygen species (ROS), thereby preventing oxidative stress-induced neuronal damage. Cheng et al. (2021) and other studies have shown that exogenous irisin can reduce the expression of autophagy markers (LC3B) in the hippocampus and cortex of rats and affect mitochondrial activity, confirming the neuroprotective effect of exogenous irisin. Irisin also plays a vital role in other neurological diseases. Kim et al. (2023) found that irisin significantly reduced amyloid-*β* (A*β*) by mediating the down-regulation of the ERK-STAT3 signaling pathway to increase the release of A*β*-degrading enzyme neprilysin (NEP) from astrocytes. The results revealed for the first time the cellular and molecular mechanisms by which exercise-induced irisin attenuates A*β*, providing a new targeted pathway for the prevention and treatment of Alzheimer’s disease.

### 4.2 Current research landscape and emerging frontiers of irisin in neurological disorders

#### 4.2.1 Alzheimer’s disease

According to the co-cited literature analysis in the above bibliometric analysis, as shown in Table 3, Professor Lourenco MV’s review article on the memory deficits of irisin in the treatment of Alzheimer’s disease was cited most frequently, which can accurately reflect the current research hotspots. Combined with keyword co-occurrence analysis, the frequency and centrality of the top five keywords, such as “Alzheimer’s disease,” indicate that this disease is undoubtedly a current research hotspot.

Alzheimer’s disease is a neurodegenerative disease that mainly affects memory, and there is currently no cure. Physical exercise is associated with cognitive benefits and reduced risk of Alzheimer’s disease. Irisin produced by physical activity has been shown to regulate synaptic function, regulate autophagy, inhibit cell death pathways, reduce oxidative stress and neuroinflammation, resulting in neuroprotective effects, and has been shown to have a good effect on improving the cognitive impairment of Alzheimer’s disease and other neurodegenerative diseases (Bellettini-Santos et al., 2023; Jaberi and Fahnestock, 2023).

Firstly, irisin can reduce the deposition of A*β* protein to protect neurons and reduce the damage of cognitive impairment. Nogueira Godinho et al. (2022) also found that high-intensity interval training (HIIT) may prevent cognitive impairment caused by A*β*42 infusion by regulating the PGC-1α-FNDC5-BDNF pathway, which is related to the increased expression of FNDC5, integrin subunit *β*5 (ITGB5) and PGC-1α in skeletal muscle. Secondly, irisin can also regulate the inflammatory response and improve the plasticity of neurons. Exercise can reduce AD risk by regulating muscle cytokine production (such as irisin and BDNF). These muscle cytokines can stimulate hippocampal neurogenesis, regulate inflammation, and reshape intestinal flora (GM) to increase the production of beneficial bacteria and short-chain fatty acids (SCFA). These effects may synergistically improve AD cognition, neuroplasticity, and gut-brain interaction (Cutuli et al., 2023). In the study of Lourenco et al. (2020), the direct correlation between irisin, BDNF, A*β*42, and cognitive status further indicated that cerebrospinal fluid irisin and BDNF may be valuable biomarkers for AD drug or non-drug intervention. In an AD mouse model, sports training-mediated hippocampal neurogenesis is associated with similar BDNF and FNDC5 expression induction, which contributes to improving cognitive function (Choi et al., 2018). These mechanisms make irisin a new research direction for interventions to prevent cognitive decline and AD progression.

Although the neuroprotective effect of irisin in AD has received extensive attention, its mechanism and clinical translation remain significantly controversial. The “gut-brain axis” hypothesis relies on correlational evidence (such as changes in gut microbiota and short-chain fatty acids), but the blood–brain barrier permeability of SCFA and its causal relationship with cognitive improvement are not yet clear. Some studies even found a negative correlation between SCFA levels and cognition, challenging the consistency of existing theories (Paniagua et al., 2025). In addition, the reliability of cerebrospinal fluid irisin as a biomarker is interfered by exercise level, specificity of detection methods, and reverse causality (cognitive decline leads to a reduction in irisin). In animal models, significant hippocampal neurogenesis may be overestimated in humans due to disputes over neurogenic capacity and differences in patient compliance. It is necessary to clarify the boundary between central and peripheral effects of irisin through conditional gene models, high-specificity detection tools and stage-adaptive clinical trials, and to reassess its treatment window and long-term safety.

#### 4.2.2 Cerebral ischemia

In the keyword cluster analysis, the # 3 Cerebral ischemia with the cluster size and the cluster average contour value ranking fourth suggests that the effect of irisin on cerebral ischemia is also a research hotspot in the field of nervous system diseases.

First, irisin can protect the blood–brain barrier from damage and reduce brain edema after focal ischemia/reperfusion injury (Guo et al., 2019). Moreover, irisin can effectively activate the classic Notch signaling pathway, thereby alleviating inflammation caused by ischemia, inhibiting neuronal apoptosis, and significantly improving neurological dysfunction (Jin et al., 2019). Secondly, irisin can also reduce neuronal damage and neurological damage induced by middle cerebral artery occlusion (MCAO), which is mediated by the down-regulation of the TLR4/MyD88/NF-κB pathway. It may also be that irisin protects neurons from damage by activating Akt and ERK1/2 signaling pathways (Li et al., 2017; Yu et al., 2020).

Currently, in the research of irisin in the field of cerebral ischemia, the focus is mainly on the mechanisms and signaling pathways. Future research needs to prioritize resolving three core controversies: (1) Clarify the cell-specific targets of irisin in the neurovascular unit; (2) Establish a standardized dosing regimen to coordinate the comparability across studies; and (3) Explore its synergistic or antagonistic effects with existing thrombolytic/anti-inflammatory therapies, rather than demonstrating a single mechanism in isolation. Only by breaking through these methodological limitations can we accurately determine the clinical value of irisin in the treatment of cerebral ischemia.

#### 4.2.3 Parkinson’s disease

Parkinson’s disease (PD) is a common neurodegenerative disease. It is generally believed that mitochondrial dysfunction is one of the pathogenesis of Parkinson’s disease (Westbroek et al., 2011). Recent studies have found that exogenous irisin has neuroprotective effects, and these effects are mainly achieved by activating the Akt signaling pathway and ERK1/2 signaling pathway. Irisin can alleviate apoptosis and oxidative stress in PD models, inhibit mitochondrial rupture, and promote mitochondrial respiration and biogenesis (Zhang et al., 2023). In addition, another study found that irisin prevented pathological synaptophysin-induced neurodegeneration in a mouse model of *α*-presynaptic fiber (PFF) in sporadic Parkinson’s disease. Therefore, targeting irisin is a new direction for the prevention, control, and treatment of Parkinson’s disease, which may be the preferred or adjuvant treatment strategy to alleviate the development of neurodegenerative diseases.

#### 4.2.4 Supplementary neurological disorders in irisin research

First of all, irisin has a positive effect on acute anxiety disorder (panic attack). Relevant research results (Jodeiri Farshbaf et al., 2020) have shown that direct injection of irisin into the hippocampus of male mice can partially prevent anxiety-like behavior caused by acute stress. Secondly, short-term administration of relatively high doses of quercetin can enhance mitochondrial biogenesis, improve mitochondrial and neuronal function adaptation through the PGC-1 pathway, and increase the expression of BDNF in the hippocampus, thereby reducing memory impairment after exposure to hypobaric hypoxia (Liu et al., 2015). In addition, irisin therapy can be used as a potential strategy for anti-epileptic oxidative stress injury. Cheng et al. (2021) confirmed that exogenous irisin has significant anti-oxidative stress and neuroprotective effects in kainic acid-induced status epilepticus.

Sports can enhance synaptic connections by promoting signal transduction of irisin and showing neuroprotective and antidepressant effects (de Oliveira Bristot et al., 2019). Another study showed that short-term subcutaneous injection of irisin in young, healthy mice showed significant antidepressant and anti-anxiety effects, and there was no gender difference (Pignataro et al., 2023).

Although current research has revealed the potential roles of irisin in anxiety disorders, epilepsy, and neuroprotection, in the future, it is necessary to analyze cell-specific targets through cross-scale research (such as single-cell sequencing and metabolomics), and optimize models for the elderly and those with comorbidities. In this way, the isolation of mechanisms can be overcome, and the precise therapeutic value of irisin in complex nervous system diseases can be established.

## 5 Conclusion

In recent years, with a deeper understanding of irisin, scholars have also widely explored and studied its mechanism of action in the nervous system. Irisin can regulate the development of neurons, inhibit pro-inflammatory cytokines, enhance anti-inflammatory cytokines to exert anti-inflammatory effects and protect neurons from oxidative stress damage by regulating mitochondrial activity. These mechanisms provide many research bases and therapeutic targets for the prevention, treatment, and prognosis of neurological diseases such as cerebral ischemia and AD.

In summary, although a large number of studies have discussed the effects of irisin on the nervous system from animal experiments and clinical applications, it still needs to reach the goal of its actual clinical application. In the future, further research can be carried out from the following aspects: Firstly, the current research on irisin in neurological diseases is limited to common diseases such as stroke and AD, and its role in other neurological diseases can be explored in the future. Secondly, recent studies have suggested that FNDC5/irisin can be used as a new drug to activate autophagy and protect the nucleus pulposus from aging and apoptosis (Zhou et al., 2022). In a review article by Anderzhanova and Voronina (2023), researchers believe that irisin activates astrocyte αv*β*5 and promotes matrix metalloproteinase-9 secretion and BDNF signal activation by positively regulating autophagy. Therefore, future research on the links between irisin and other targets or signaling pathways, such as autophagy, will improve our understanding of the mechanisms underlying the treatment of neurological diseases. Third, some of the article’s conclusions, opinions, and explanations are inevitably based on results obtained using animal models and laboratory rodents (Cutuli et al., 2023). Therefore, further research on human subjects is needed in the future. Fourth, most of the current research articles have proved that physical exercise may increase the level of irisin in the blood (Jandova et al., 2021). Still, the specific types of physical exercise have not been thoroughly studied on the level of irisin production, which is not conducive to the application of irisin in clinical diseases. Finally, irisin is still in its infancy as a potential treatment strategy for neurological diseases such as Alzheimer’s disease. The current research urgently needs to build a systematic research framework from the following three dimensions. First, at the molecular level, the focus should be on elucidating the specific transport mechanism of irisin across the blood–brain barrier and elucidating the precise interaction patterns with different receptor subtypes in the FNDC5/BDNF signaling axis; Second, at the translational medicine level, peripheral irisin levels post-exercise should be correlated with cognitive outcomes (e.g., MMSE scores) to identify CNS-linked biomarkers. Standardized aerobic protocols (e.g., 30 min sessions, 4x/week) can quantify exercise-irisin dynamics through longitudinal trials. Third, at the clinical application level, it is recommended to conduct multicenter clinical trials focusing on monitoring the dose–response relationship between cognitive improvement (assessed by ADAS-Cog scores) and reduced brain A*β* deposition at weeks 8, 16, and 24 of treatment. This will facilitate the establishment of a precision medication strategy featuring “low-dose maintenance for early intervention and stepwise dose escalation for mid-to-late disease stages.”

It is believed that in the future, irisin can be better used to improve neurological diseases, explore its mechanism of action, and provide clinicians with new means of diagnosis and treatment.

## Data Availability

Publicly available datasets were analyzed in this study. This data can be found here: https://webofscience.clarivate.cn/wos/author/search.
